# Animal Models of Metabolic Epilepsy and Epilepsy Associated Metabolic Dysfunction: A Systematic Review

**DOI:** 10.3390/ph13060106

**Published:** 2020-05-26

**Authors:** Uday Praful Kundap, Yam Nath Paudel, Mohd. Farooq Shaikh

**Affiliations:** 1 Research Center of the University of Montreal Hospital Center (CRCHUM), Department of Neurosciences, Université de Montréal, Montréal, QC H2X 0A9, Canada; uday.kundap@umontreal.ca (U.P.K.); yam.paudel@monash.edu (Y.N.P.); 2 Neuropharmacology Research Strength, Jeffrey Cheah School of Medicine and Health Sciences, Monash University Malaysia, Selangor 47500, Malaysia

**Keywords:** metabolic epilepsy, animal model, mitochondrial dysfunction, metabolic genes, translational research

## Abstract

Epilepsy is a serious neurological disorder affecting around 70 million people globally and is characterized by spontaneous recurrent seizures. Recent evidence indicates that dysfunction in metabolic processes can lead to the alteration of neuronal and network excitability, thereby contributing to epileptogenesis. Developing a suitable animal model that can recapitulate all the clinical phenotypes of human metabolic epilepsy (ME) is crucial yet challenging. The specific environment of many symptoms as well as the primary state of the applicable neurobiology, genetics, and lack of valid biomarkers/diagnostic tests are the key factors that hinder the process of developing a suitable animal model. The present systematic review summarizes the current state of available animal models of metabolic dysfunction associated with epileptic disorders. A systematic search was performed by using the Preferred Reporting Items for Systematic Reviews and Meta-Analyses (PRISMA) model. A range of electronic databases, including google scholar, Springer, PubMed, ScienceDirect, and Scopus, were scanned between January 2000 and April 2020. Based on the selection criteria, 23 eligible articles were chosen and are discussed in the current review. Critical analysis of the selected literature delineated several available approaches that have been modeled into metabolic epilepsy and pointed out several drawbacks associated with the currently available models. The result describes available models of metabolic dysfunction associated with epileptic disorder, such as mitochondrial respiration deficits, Lafora disease (LD) model-altered glycogen metabolism, causing epilepsy, glucose transporter 1 (GLUT1) deficiency, adiponectin responsive seizures, phospholipid dysfunction, glutaric aciduria, mitochondrial disorders, pyruvate dehydrogenase (PDH) α-subunit gene (PDHA1), pyridoxine dependent epilepsy (PDE), BCL2-associated agonist of cell death (BAD), Kcna1 knock out (KO), and long noncoding RNAs (lncRNA) cancer susceptibility candidate 2 (lncRNA CASC2). Finally, the review highlights certain focus areas that may increase the possibilities of developing more suitable animal models and underscores the importance of the rationalization of animal models and evaluation methods for studying ME. The review also suggests the pressing need of developing precise robust animal models and evaluation methods for investigating ME.

## 1. Introduction

Metabolic abnormalities (ME) causing high brain activity are associated with an increased risk of epilepsy development in affected individuals. ME is caused by an array of toxic or metabolic diseases, such as mitochondrial dysfunction, alteration of intracellular osmolytes, accumulation of toxic substances, and a decrease of substrates that are crucial for internal membrane function or cellular metabolism [1]. All these factors combined result in a compromised efficacy to supply energy in the brain area, leading to excitability of the neuronal cells and producing epileptic seizures [2]. Moreover, the novelty of this research would be the study of how metabolic dysfunction can contribute to seizures and exacerbate related sequalae such as neuronal loss and related complications [3].

**Figure 1:**
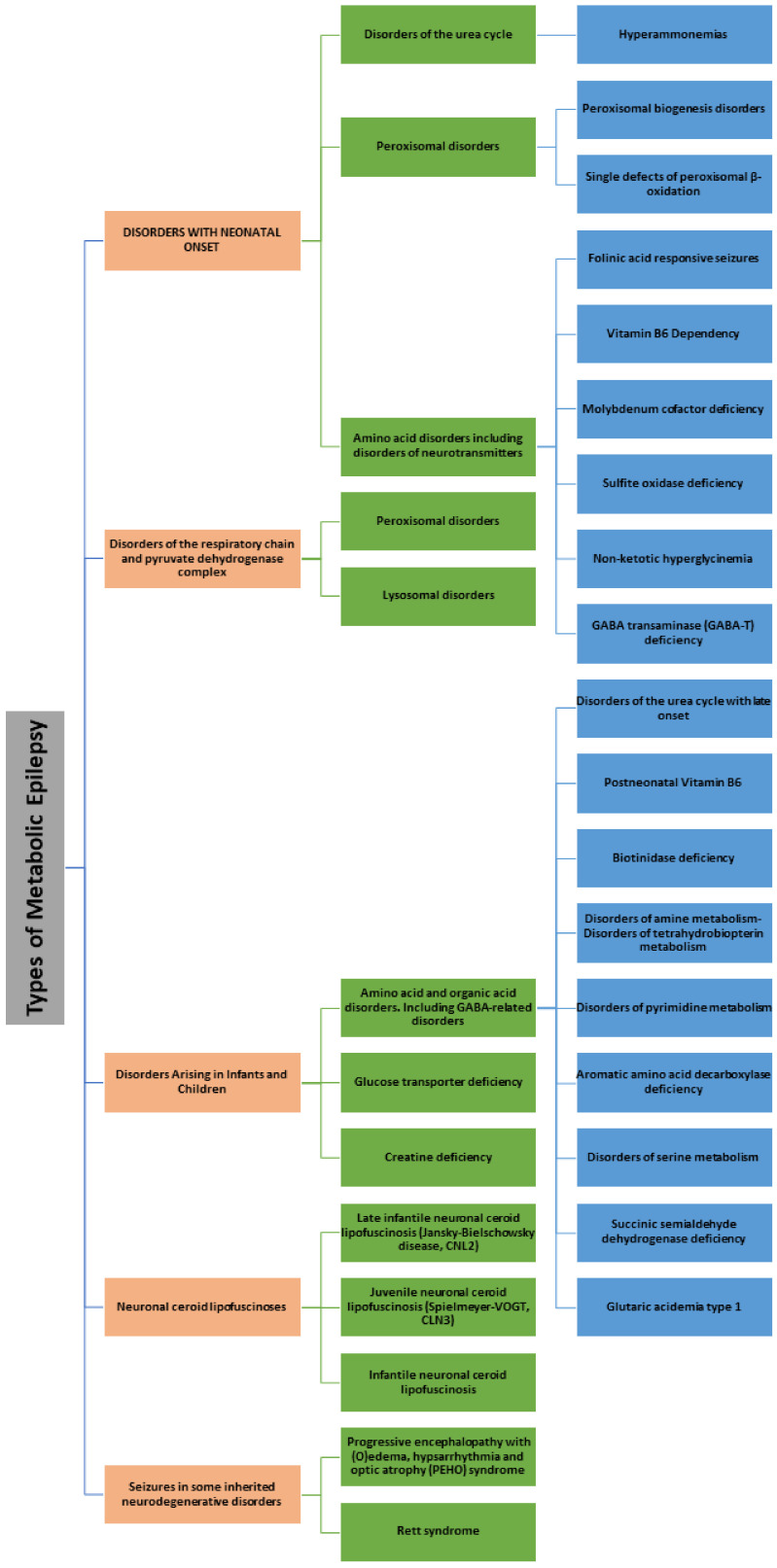
Types of Metabolic Epilepsy.

According to the International League Against Epilepsy (ILAE), ME is classified based on deficiency syndrome and disorder related to mitochondria or metabolism [4] (Figure 1). Biotinidase deficiency or “Holocarboxylase synthetase deficiency” is a condition whereby the body is not able to utilize biotin properly [5]. The impairment of certain enzymes that are biotin dependent are categorized under a group of disorders known as “multiple carboxylase deficiencies” [6]. Cerebral folate deficiency (CFD) is a neurological syndrome associated with a low cerebrospinal fluid (CSF) concentration of 5-methyltetrahydrofolate (5MTHF) in the presence of normal peripheral folate metabolism [7]. Moreover, CFD might result in cerebellar ataxia, epilepsy, dyskinesia, psychomotor retardation, and spastic diplegia [8]. Disturbances in folate transport, which might be due to increased folate turnover within the central nervous system (CNS), may also lead to CFD [9]. In a majority of the CFD cases, the etiology remains elusive, however there is an increased understanding about the key role of mutation in folate receptor 1 (FOLR1) gene in CFD [10]. Further, folate receptor auto-antibodies suggests that CFD may be caused by the blocking of folic acid transport into CSF [8]. Creatine disorders are comprised of three defects, namely reduced creatine production in guanidinoacetate methyltransferase (GAMT), deficiencies of arginine glycine amidino transferase (AGAT), and decreased transport of creatine into the brain [11]. Epilepsy is associated with GAMT deficiency, which positively responds to the treatment which is substitutive with creatine monohydrate [12]. Folinic acid responsive seizures are diagnosed as an increase in monoamine metabolite in CSF, however, their genetic cause remains elusive [13]. Neonatal epileptic encephalopathy might be a cause of folinic acid responsive seizures and is treatable as the patients with this type of seizures respond well to pyridoxine therapy [14].

Mutations in solute carrier family 2 member1 (SLC2A1) gene are the cause of glucose transporter 1 (GLUT1) deficiency syndrome and results in the improper transportation of glucose into the brain [15]. A common inborn error of energy metabolism are a mitochondrial respiratory chain disorder. Tissues with a high energy requirement are usually affected by these disorders, which are frequently observed in childhood cerebral involvement and often leads to seizures [16]. Prominent myoclonic seizures are a common characteristic feature of numeral mitochondrial disorders together with Alpher’s syndrome, myoclonic epilepsy with ragged red fibers (MERRF), mitochondrial encephalopathy with lactic acidosis, and stroke-like episodes (MELAS) [17,18]. A group of inherited diseases where either peroxisomal function or one or more peroxisome biogenesis functions are disrupted are known as a peroxisomal disorder [19]. Pyridoxal 5′-phosphate is the naturally active form of pyridoxine which is converted by a series of enzymes involving pyridoxamine phosphate oxidase (PNPO) [20]. Decreased levels of pyridoxal 5′-phosphate in the CSF along with epilepsy are usually associated with PNPO [21].

Human ME constitutes a ranges of clinical, electrical, and behavioral demonstrations [22]. The selection or development of an animal model system is determined by several crucial factors, such as the type of epilepsy to be modelled, including the reason to be studied, acquaintance, and suitability, because of the large variety of pathological mechanisms involved in ME [23].

**Figure 2:**
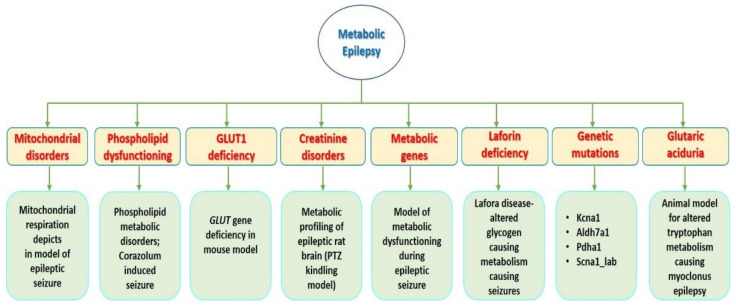
Animal models of ME and epilepsy associated metabolic dysfunctions: This figure demonstrates the overall studies of animal model related to ME with different multi approach considered in the current review.

Overall, 23 animal model studies that were mainly focused on different aspects of ME are reviewed in the current systematic review. Current study discusses the metabolic alterations studied in animal models that are associated with epilepsy. The major types of models are described with the use of animals to study metabolic disorders causing epilepsy or related disorders of CNS, and are further classified as follows: biotinidase and holocarboxylase synthase deficiency, CFD, creatine disorders, GLUT1 deficiency, folinic acid responsive seizures, mitochondrial disorders, peroxisomal disorders and pyridoxine-dependent epilepsy, and genetic knock out (KO) models (Figure 2).

Developing a suitable animal model that can recapitulate the clinical features of human ME is challenging as well as interesting [24]. The development of an animal model is complicated due to the specific nature of various symptoms, lack of validated biomarkers and objective diagnostic tests, the primary level of the specific neurobiology, and genetic factors [25]. Developing suitable animal models of ME will open a window of opportunity in this domain as well as strengthen our knowledge of the complex pathophysiology, mechanism, and therapy for the treatment of ME [26]. Herein, we systematically review the current state of several available pre-clinical models of ME and other related alterations. With respect to the reviewed literature, the authors consider the probable areas of interest that might intensify the likelihood of generating more valuable models, at least for ME with related symptoms, and for explicit methods where animal models are used [27]. To the best of our knowledge, no earlier reviews have systemically considered the available animal models that are used to investigate ME and associated alterations. Hence, the current review in a systemic way provides readers with a precise summary of available animal models that have been used to investigate several aspects of metabolic alterations associated with, but not necessarily responsible for epileptic disorders. In addition to a single read, this systematic review would strengthen the understanding of readers of the importance of developing animal models of human ME. Moreover, the current systematic review encourages that ME can also be modeled in a range of species, including zebrafish, tilapia, and drosophila [28,29].

## 2. Results and Discussion

**Figure 3:**
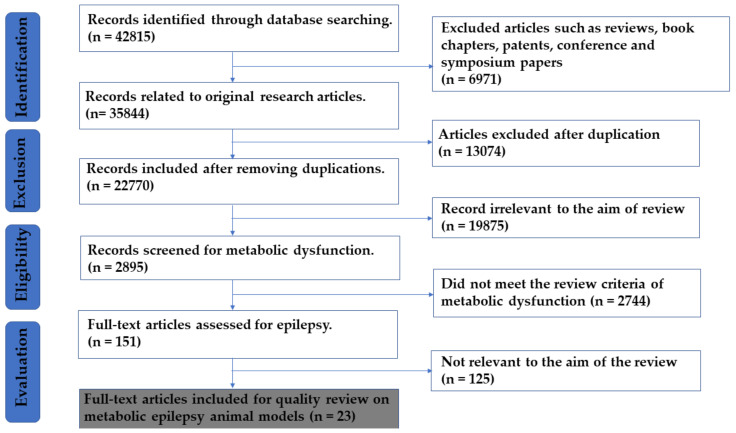
Study evaluation PRISMA flow chart for selection of articles.

Exploring the selected database with the specific key words mentioned above in the methodology yielded 42,815 records. After screening, the total number of articles excluded were 42,792, and thereof: (a) 6971 abstracts, books, chapters, patents, symposiums, oral and poster presentations, and conferences; (b) 13,074 duplicate articles were excluded; (c) 22,619 articles that did not meet review criteria, review articles, and articles irrelevant to the aim of the review were also excluded. A further 23 articles were compiled, included, and described in Table 1 and discussed in the systematic review forum (Figure 3).

The 23 articles discussed herein consist of original research articles, pre-clinical studies, and research reports which provide information of the available animal models for ME. In addition, the number of articles found were limited to the type of metabolic study conducted using specific animal species. During our analysis, we observed that, to date, only mice and rats were used to study ME. However, a few human studies have also been recorded. As the precise mechanism underlying how metabolic dysfunction can lead to epilepsy is still unknown, it is very challenging to mimic the same clinical conditions in animal settings. We attempted to summarize all the article and describe the main significant feature of the study to highlight the role of metabolic disorder in epileptogenesis. Nevertheless, there are very few articles available. Moreover, we tried to classify them according to the available classification scheme mentioned by ILAE. Books with different chapters on “epilepsy in children, newborns, and inborn errors of metabolism” [30,31,32], as shown in Table 1, Figure 1, Figure 2, and research reports and retrospective studies were included despite their limitations and scope [32].

### 2.1. Metabolic Genes Responsible for Epilepsy in the Obese Rat

Mounting evidence has suggested that obesity may be associated with disorders of the neural pathway [56]. Obesity is highly comorbid with neurological disorders [57]. In a clinical trial of pediatric populations diagnosed with epilepsy, body mass index (BMI) has been found to be elevated in epileptic populations as compared to normal control [58]. Anti-epileptic drugs (AEDs) associated weight gain have also been reported in many studies. In an experimental study, lithium and pilocarpine induced status epilepticus (SE) in female Wistar rats, causing weight gain and obesity [59]. Moreover, findings are emerging which report that patients under valproic-acid therapy have greater chances of the progression of metabolic disorders [60]. Various genes associated with metabolic dysfunction and the neuroendocrine regulation of obesity might contribute to neurological disorders like epilepsy [61]. Intracellular glucocorticoid metabolism in the brain has been regulated by 11β-hydroxysteroid dehydrogenase Type 1 (Hsd11b1) [62]. In rodents and humans, the upregulation of Hsd11b1 gene in adipose tissue and the brain is associated with obesity, metabolic dysfunction, and neuronal degeneration [63]. Glucocorticoid receptors (Nr3c1) have an affinity towards cortisol, which is present in several brain regions and peripheral tissues, and play a crucial role in regulating negative feedback mechanisms of the metabolic function, hypothalamus-pituitary-adrenal axis, and many other physiological processes [33]. In spite of the fact that the comorbidity of obesity and epilepsy has been recently studied [58], a couple of studies have shown the basic mechanisms relating to the association between weight gain and epilepsy [59].

The genes linked with neuroendocrine function or metabolism that regulates obesity (Nr3c1, Hsd11b1, Kcnj11, Abcc8, Drd2, NPY, Mc4r, Lepr, and brain derived neurotropic factor (BDNF) were determined [64]. The expression levels of Hsd11 were significantly upregulated in animals with epilepsy at 24 h post-SE, and the decrease in the expression level takes place at 10 days and one month. As the Hsd11b1 level increases (through a negative feedback mechanism of increased levels of intracellular cortisone), the level of Nr3c1 is down-regulated [65]. It has been reported that, during epileptogenesis, glucocorticoid metabolism is changed, and this might be due to alterations in metabolic gene expression of glucocorticoid, [33].

Downregulation of BDNF has a detrimental effect on glucocorticoids. It was observed that Abcc8 mRNA levels were downregulated and Kcnj11 levels tend to increase by two months post-SE. The study reported no significant difference in the hippocampal mRNA expression level of some of the genes like MC4r, Drd2, NPY, or BDNF in the control and epileptic animal. The results of Lepr gene mRNA levels were too low in the hippocampus to perform the analysis. After Pilocarpine administration in rats, they become rigorously obese and demonstrated substantial differences in the hippocampal expression level of genes that are involved in energy metabolism and glucocorticoid regulation. Herein, authors hypothesized that feedback loops regulating energy metabolism and dysregulation of neuroendocrine mechanisms in the hippocampus might be due to epileptogenesis [33]. The animal model utilized herein can be further used to study various parameters related to gene expression of metabolic dysregulation in epilepsy with obesity.

### 2.2. Metabolic Profiling of Epileptic Rat Brain in PTZ Kindling Model

Globally, over 70 million people suffer from epilepsy [66,67], and up to one third of people with epilepsy do not respond to mainstream AEDs [68]. The PTZ kindling model is among the most frequently used models to induce epilepsy and is characterized by an increased susceptibility to seizures [34,69]. Seizures are known to increase blood flow, overall brain metabolic rate, flux through glycolysis, and the tricarboxylic acid (TCA) cycle [70,71]. These metabolic alterations are thought to be a result of the increased adenosine triphosphate (ATP) demand during a seizure [70]. Over the years, very few animal model prototypes have been developed to study the basic mechanisms of metabolic alteration leading to epilepsy. During seizures increase in ATP demand is thought to be due to metabolic alterations [72]. Amino acid metabolism has been widely studied in recent years, and studies suggest that PTZ kindling favored the uptake of specific amino acids, namely leucine, but demolished the process of transamination reaction, resulting into glutamate production [73]. An earlier reported study performed proton nuclear magnetic resonance (^1^H NMR) spectroscopy in combination with multivariate data analysis on cellular extracts from four brain regions with the aim to identify metabolic changes that occur following a seizure. This metabolomics approach has been successfully applied in order to the study several neurological disorders, such as Batten disease, Huntington’s disease, and spinocerebellar ataxia [34]. This study utilizes unique approach in order to get deep insights into the changes in global metabolic rate of glutamate and other essentials transmitters after seizures [74]. The study also reported decreased levels of *N*-acetyl aspartate (NAA) in PTZ-kindled animals, and also reduced in diseases where neuronal loss takes place [75]. The same result was found in kainic acid-induced seizures and related alterations [76,77].

### 2.3. Mutation of Ubiquitin Ligase (Ube3a Phenotype) Causes Angelman Syndrome in Mice-a Rare Genetic Epileptic Neurodegeneration

Angelman syndrome (AS) is an uncommon neurodegenerative disorder characterized by seizure disorder with a specific electroencephalogram (EEG) [78]. AS is detected in one out of every 12,000–20,000 population. Severe delays in developmental and childhood epilepsy are involved in AS which is considered as one of the important genetic syndromes [79]. Moreover, the disorder exhibits some specific features, such as difficulty in learning, ataxia, and subtle dysmorphic facial features [80]. Moreover, the disorder is triggered due to multiple genetic abnormalities, including in the chromosome 15q11-13 region [81]. Patients with AS demonstrate problems in controlling epileptic seizures and are often noncompliant to many prescribed medications involving several seizure types [35]. AS occurrs in humans during the maternal deficiency of ligase protein and it was observed that an E6-AP ubiquitin ligase (mouse gene UBE3A/humane/ube3a) supports the deprivation of p53. The study conducted by Jiang et al. in 1998 reported that mutation in Ubiquitin-protein ligase E3A (Ube3a) phenotype of mice with maternal insufficiency (m−/p+) replicates to human AS with induced seizures and demonstrates dysfunction in motor coordination and deficits in learning [36]. Long-term potentiation (LTP) was significantly decreased in mutant mice which suggests that it might be abnormal in AS. To confirm the model for AS, the cytoplasmic accumulation of p53 was reported to be upregulated in postmitotic neurons in mice with AS. Mice demonstrated abnormal behavior and ataxia according to EEG. As well as the size of skeletal bone, brain weight was observed to be reduced, and these features are similar with human AS patients. One of the major metabolic disorders observed was the late onset of obesity which might be due to an increase in the cytoplasmic profusion of p53. The study highlighted that null mutation for Ube3a mice at maternal deficiency clearly recapitulates the phenotype of human AS. E6-AP protein might disturb the metabolism of p53 in postmitotic neurons, which may upregulate the concentration of p53. The study also suggested that the development of a mouse model will allow the opportunity to categorize other protein targets to E6-AP, which are supposed to regulate metabolic disorders that might lead to AS and epilepsy [36].

### 2.4. Metabolic Features in Repetitive Seizures

According to ILAE, repetitive seizures are those exhibiting continuous seizure episodes observed within 24 h [4]. The prevalence of seizures is comparatively higher in children as compared to the young and adult population. There is an increased understanding that early life seizures might trigger long-lasting changes [82]. Childhood seizures, either repetitive or prolonged, might lead to damage to the neuronal metabolic pathway as well as problems in learning and memory like Alzheimer disease [83]. Flurothyl (FL) was used to induce repetitive brief seizures in mature and immature rats. The rationale behind this study was to suggest metabolic activity (2-deoxyglucose labeling) and markers of neuronal activity (c-fos mRNA expression) in repetitive epileptic immature and mature animals. This study sheds light on the extent of damage to metabolic activity and gene expression that occurred during repetitive seizures in the brain. The study also well reported that c-fos mRNA expression level was highly expressed in the major areas of immature rats as compared to the adult rats. Repetitive seizures resulted in lower 2-deoxyglucose labeling in many regions of the brain. Neuronal activity patterns and seizure behavior are significantly observed in the immature rats as compared to the mature rats. It is worth noting that the 2-deoxyglucose labeling technique discussed in the study is not sensitive enough to measure metabolic activity that could unravel the distinct FL seizure-related changes [23]. The study clearly highlighted the importance of developing a suitable animal model that would allow to determine the effects and metabolism in animals of repetitive epilepsy and to correlate these variations to those in the adult animals.

### 2.5. Epilepsy and Metabolic Dysfunction in a Mouse Model-Glut Deficiency (G1D)

Glucose has a profound role in brain growth and neural excitation as glucose is the major source of carbon and energy for the brain [84]. Most of the carbon required is supplied by the brain to generate acetyl-coenzyme A (acetyl-CoA), which is one of the vital steps in myelin synthesis [85]. Further metabolism of acetyl-CoA is responsible for neurotransmitter production via the TCA cycle. Impairment of glucose transportation into the brain is caused due to GLUT1 deficiency syndrome. It is related more with hyper-excitability rather than hypo-excitability which leads to seizure activity in patients [86]. Despite this, our knowledge of brain metabolism during Glut deficiency (G1D) remains limited. Currently available mouse models of G1D have broadened the knowledge and allowed to investigate the energy failure or metabolism of the brain during the disease. This study has provided a novel aspect of cerebral metabolism and hyper-excitable circuit during GLUT1 deficiency by identifying key metabolic and electrophysiological features of transgenic mice, i.e., antisense GLUT1 [37]. Moreover, this study clearly described the somatic metabolic features of antisense mice, general phenotype, and electrophysiological changes of GLUT1 deficiency syndrome causing epilepsy like disorders. The study also attempts to replicate the most common clinical phenotypes of human including seizures and predominantly movement disorders. The results generated herein provided the novel perspectives related to fatty acid synthesis, flux from fatty acids to triglyceride, anaerobic glycolysis, and cholesterol esters in GLUT1 deficiency as these mechanisms are plausibly responsible for epileptogenesis [37].

### 2.6. Mitochondrial Respiration Deficits in Rat Epilepsy Model

Metabolism is simply described as the biochemical progressions taking place within the living organism in order to sustain life [16]. Impairment of metabolic function accounts for the cause of several neurological disorders. Among the several known functions, the generation of ATP is the prime function of mitochondria [87]. The primary purpose of mitochondria is to generate reactive oxygen species (ROS) and the production of ATP, but they also exhibit an ability to induce seizures. This is mainly because the energy requirement during the seizure and mitochondrial sensitivity might lead to oxidative damage [88]. A wide variety of mutations in nuclear genes or mitochondrial DNA progressing to the impairment of mitochondrial respiratory chain or synthesis of mitochondrial ATP have been associated with epileptic disorders [3]. In addition, mitochondrial malfunction leads to neuronal cell death, which is an important feature of TLE [87]. In the model of temporal lobe epilepsy (TLE), it was reported that mitochondrial respiration might be impaired by oxidative damage to electron transport chain (ETC) enzymes, leading to the increased generation of mitochondrial ROS. The aim of this study was to determine if variations in cellular bioenergetics occur using the real-time analysis of depletion in oxygen level in the mitochondria and glycolytic rates in an animal. The study hypothesized that epileptogenic injury initiated the amplified steady-state levels of ROS which might result in impaired mitochondrial respiration [38]. The study validated that ROS mediated metabolic problems exists in experimental TLE. It is well reported that respiration defects in mitochondria occur during TLE and ROS experimental models, which might mechanistically contribute to these defects. The finding of the study provides deep insights about the novel perspectives for evaluating cellular metabolism during the complete time frame of disease progression [38].

Oxidative stress is a well-known player in the pathogenesis of neurological disorders [89]. The mitochondria are considered as the prime source of ROS causing oxidative stress. Earlier study has demonstrated the precise role of NADPH oxidase (NOX) enzymes in generating ROS [90]. The cellular function by NOX enzymes in CNS has a pathological effect on many cell types, but the precise mechanism is not yet well understood [91]. The systematic review of the effect of antioxidant compounds on neuropathological alterations represents psychotic-like symptoms resembling the human first psychotic episode. Oxidative stress and redox dysregulation have a negative impact on the CNS that might initiate the progression of healthy mental status to a psychotic state [27]. An increase in substantial levels of oxidative stress biomarkers and reduced levels of antioxidants are reported to be present in epileptic subjects. The increase in the generation of ROS has been documented to be responsible for inducing epilepsy by recurrent seizures as well as by mitochondrial dysfunctions [28].

Autophagy is a catalytic process which plays a role in maintaining cellular homeostasis by the degradation of cytoplasmic macromolecules and organelles. Cell death is reported to be associated with excessive autophagy [29]. Further, a huge number of studies have reported that autophagy might play a crucial role in several neurological disorders. There are very few studies describing the putative role of autophagy in epilepsy [92]. NOX-induced oxidative stress plays a major role in inducing autophagy, but the role of NADPH-mediated autophagy in epilepsy is still unknown. The study suggests that PTZ kindling induces the production of ROS and peroxidation of lipids, which causes mitochondrial injuries and the activation of NOX [93].

### 2.7. A Rat Model of Pilocarpine-Induced Epilepsy with an Abnormality in Metabolic Connectivity

Functional connectivity (FC) between brain regions is mapped using functional magnetic resonance imaging (fMRI) on the basis of blood-oxygen-dependent (BOLD) signals that oscillate synchronously [94]. The activation of membrane phospholipases, proteases, and nucleases which cause debasement of membrane phospholipids, proteolysis of cytoskeleton proteins, and protein phosphorylation is the main feature of long term seizure progression [95]. This alteration marked the major liberation of free fatty acids (FFA), mainly free arachidonic acid, lipid peroxides, and radical free ions [96,97]. Some models of PTZ kindles have stated the changes in oxidative defense mechanism in frontal cortex, hippocampus causing increase in FFA, glutathione peroxidase, and superoxide dismutase increase [98]. The over utilization of brain glucose increases after long term seizure activity, which causes neuronal damage [99]. Information regarding human brain metabolism and disease state is provided by the rationality of signals among the brain. fMRI alone cannot accurately assess the effective connectivity (EC) across the brain [39]. The study proposed an innovative way to measure EC which is labeled as metabolic connectivity mapping (MCM), that assimilates one-directed FC with local energy metabolism from fMRI and positron emission tomography (PET) data acquired simultaneously [39]. The graphical approaches of theoretic means were used to evaluate complex brain connectivity and to measure regional abnormal brain metabolism and topologic features. The connectivity of abnormal metabolism was demonstrated in the rat model of pilocarpine-induced epilepsy [100]. It was reported that the functional correlation was significantly different along with graphical theoretic properties between the epileptic rats and control groups. The involvement was found particularly in the amygdala and entorhinal cortex. The study also unraveled the connections that are abnormal and involved in the left insular cortex as well as in the left amygdala by using threshold-free network modeling [101]. The topological properties and brain network modeling can provide vital information about various brain disorders and functional connectivity abnormalities in diseases such as epilepsy. Furthermore, the results obtained from rodents and other models indicate that metabolic functional brain network analysis can be a useful tools for pre-clinical research using rat brain models that can produce the hallmarks of different human brain dysfunctions like epilepsy [39].

### 2.8. Epilepsy Due to Metabolic Dysfunction via Adiponectin Deficiency

Genetic diseases concerning disorders of metabolism are mainly present in the form of inborn errors. Genetic diseases normally occur in the newborn and in infants but can even occur during adulthood [102]. Metabolic syndromes have a detrimental effect on the CNS, and recent findings suggest that obesity rates are higher in children with metabolic epilepsy. Adipose tissues are responsible for the secretion of adiponectin which regulates lipid and glucose metabolism in the brain and peripheral organs of our body [103]. A frequent symptom of seizures, no specific EEG pattern, or seizure types constitute the main characteristic features of inborn errors of metabolism [104]. Insufficiency of adiponectin leads to metabolic symptoms which are characterized by hyperlipidemia, impaired glucose tolerance, obesity, cardiovascular morbidity, insulin resistance, and neurodegenerative disorders [105]. Adiponectin can act as a protective agent against ischemic brain injury and other neurological disorders via interfering with inflammatory pathways and endothelial functions [106].

This study mainly focused on whether seizures associated with brain injury would aggravate metabolic syndrome due to adiponectin insufficiency. To study the relationship between epileptic seizures and metabolic syndrome, adiponectin KO mice and wild-type C57BL/6J were fed a high-fat diet (HFD). The lowest dose of kainic acid (KA) treatment was administered to induce seizures. The greater fat accumulation was reported in mice fed with HFD having adiponectin deficiency. This resulted in the accumulation of fats, impaired glucose tolerance, increased seizure severity, as well as led to hyperlipidemia and increased hippocampal pathology. The study attempted to develop an animal model replicating features of metabolic dysfunction leading to epilepsy via adiponectin deficiency. The incidence of clonic seizures was reported in 50% of HFD-fed adiponectin-KO mice. A low dose of intra-hippocampal KA resulted in severe neuronal damage and gliosis in adiponectin-KO mice [40]. There was no increment in seizure sensitivity in the normal chow-fed mice with adiponectin deficiency. This result highlighted that adiponectin deficiency might stimulate brain pathology and seizure activity due to changes in metabolic parameters. A better understanding of the precise mechanisms of how adiponectin deficiency stimulates brain pathology and seizure activity would offer a broader horizon for further model development that could assist in the prevention and treatment of epilepsy linked with metabolic syndrome [40].

### 2.9. Myoclonus Epilepsy Model: Impairment of Serotonin (5HT) and 3-Hydroxyanthranilic Acid Metabolism

Myoclonus epilepsy is characterized by continuous convulsive frequent febrile seizures, followed by non-febrile seizures, mainly unilateral and clonic of frequent SE and long duration [107]. The loss of function in mutation genes takes place inside the gene encoding cystatin B (CSTB) and might cause several neurodegenerative diseases. The cathepsin family of proteases is known as a cysteine protease inhibitor [108]. The goal of the study was to assess the amount of tryptophan and its metabolites in the brain. As well as the study evaluates the metabolism of tryptophan in serum of mice deficient with CSTB (a model system for EPM1) and progressive myoclonus epilepsy (EPM1) patients along 5-HT and kynurenine (KYN) pathways. The results demonstrate that the metabolism of tryptophan was disturbed in EPM1 along 5-HT and KYN pathways [41]. The metabolism of tryptophan by 5-HT and KYN pathways are well established, whereby tryptophan is converted to 5-hydroxytryptophan via Tryptophan-5-hydroxylase enzyme. These metabolites are further decarboxylated to form 5-hydroxy-tryptamine [109]. Homozygous mice mimicking the disruption in CSTB gene is now available and the same transgenic mice display the conditions similar to EPM1 patients pre-clinically [110]. The study also outlined that tryptophan metabolism along with 5-HT and KYN pathways are disturbed in EPM1. Disturbances of 5-HT metabolism in the brain are due to a mutation in the CSTB gene and they are not related to a decrease in the level of L-tryptophan (a 5-HT precursor). KYN pathway is well known for tryptophan metabolism and reported to be abnormal in EPM1 patients and CSTB-deficient mice [41].

### 2.10. Model for Metabolic Dysfunction during an Epileptic Seizure in Pilocarpine-Treated Rats

TLE includes all seizure types or electric firing occurring in the temporal lobe region of the brain, irrespective of the pathology and the location of the initiation of seizures [111]. It depicts that seizures are specifically arising in the mesial structure of the entorhinal cortex, hippocampus, and amygdala. TLE is the common form of epilepsy prevailing from the focal region and is recurrently unaffected by anticonvulsants, whereas in a few patients, it is progressive in nature [111]. The mechanism underlying the pathogenesis of TLE still remains poorly understood. However, there is a notion that metabolic dysfunction contributes to the pathogenesis of TLE. Functional neuroimaging studies of hippocampal tissue from TLE patients displayed a reduction in glucose consumption in seizure foci and corresponding brain structures in epileptic patients. This might be due to the disruption of oxidation or glycolytic energy metabolism. However, the study did not report any detailed record investigating the cause metabolic or mitochondrial dysfunction during neuronal activation.

On a positive note, the next study characterized mitochondrial and metabolic functions in Pilocarpine-treated hippocampal slices of chronic epileptic rat [42]. The results suggested that the NADPH recording under fluorescence light indicted the conditions of cellular energy metabolism during neuronal activation in several areas of acute hippocampal slices. In control rats, the electrical stimulations described by a brief early drop and followed by prolonged overshoot indicated an increase in NADP^+^ decline. Whereas in epileptic rats, overshoots were significantly smaller in the hippocampal cornu ammonis 1 (CA1) area. However, In TLE patients who were classified as two groups, namely Ammon’s horn sclerosis (AHS) and non-AHS, a large drop and very small overshoot were observed in the CA3, dentate gyrus, CA1, and subiculum. The metabolic dysfunction elicited in each neuron of AHS tissues represents a negative activation-dependent mitochondrial depolarization. These findings were established by applying confocal laser scanning microscopy in specific neurons of AHS tissue, indicating a negative action potential and mitochondrial depolarization-dependent activation. The neurons and glial cells in metabolic dysfunction might affect ATP homeostasis significantly and intrinsic anti-oxidative mechanisms as well. Under specific situations, this turbulence might favor the manifestation of seizures and neuronal vulnerability along with status epilepticus [42]. These findings indicated a severe dysfunction in the metabolic process during epileptic seizures in the hippocampus in humans and chronic epileptic rats. Thus, the findings suggest that cellular hypometabolism in the epileptic cells in the brain indicate mitochondrial enzyme defects in TLE.

### 2.11. Lafora Disease Model-Altered Glycogen Metabolism Causing Epilepsy

LD is caused by an interaction between two enzymes, laforin and malin (ubiquitin E3 ligase). It is characterized by the formation of weak branched polymer like glycogen (polyglucosan), also known as lafora bodies. These lafora bodies accumulate in the liver, various parts of muscular structures, neurons, and other tissues [112]. In the brain, the neuronal dendrites are overtaken by these accumulated lafora bodies and initiate neuronal degeneration followed by fatal seizures. Disrupted long-chained glycogen is accumulated in many brain cells, causing epilepsy [113]. About half of the LD cases results from mutations in the epilepsy progressive myoclonus type 2A (EPM2A) gene, which encodes laforin, a member of the dual specificity protein phosphatase family capable to release the small quantity of covalent phosphate normally present in glycogen [112].

In the study discussed herein, authors attempted to genetically block the synthesis of brain glycogen in LD mice. The mouse model of LD in this research is described as *Epm2a^−/−^* LKO model (mixed C57BL/6J and 129Sv/J). Offspring with these two genetic knockdown *Epm2a^−/−^/Gys1^+/+^* are labeled as LKO mice model whereas *Epm2a^−/−^/Gys1^+/+^* knockdown are labeled as DKO experimental mice. LKO mice are characterized by increased astrocytes and gliosis. The genetic modulation resulted in the long-term prevention of formation of laforin bodies, neurodegeneration, and seizure onset. The study postulated that glycogen synthesis is necessary for lafora body formation, leading to LD, which in turn results in progressive myoclonic epilepsy. The findings of the study also point out that lafora bodies were found to be pathogenic and the main cause of neuronal degeneration and fetal death within 10 years of the first epileptic episode. In addition, the study suggests that the animal model utilized herein is suitable for the study of metabolic epilepsy where the inhibition of glycogen synthesis prevents the formation of pathogenic lafora bodies, thus preventing epilepsy. These findings opens a novel avenues for the treatment of LD with known small molecule glycogen synthesis inhibitors [43].

### 2.12. Animal Model for Phospholipid Metabolic Disorders: Corazolum Induced Seizures

Phospholipids are the two parallel layers arranged together and lined up to form a phospholipid bilayer. Cell membranes are built up by phospholipid bilayer and play a vital role in cell function [114]. Phospholipid plays an important role in cell metabolism, cell structure, as well as functional and physicochemical characteristics [115]. Breakdown in the phospholipid membrane has been implicated in neurodegeneration [116].

One of the earlier reported studies well investigated how experimental epileptoid seizures induced by Corazolum in rodents leads to quantitative and qualitative disruptions in phospholipids, affecting the proportion between phospholipids, and changing the quotient K-the ratio between total neutral phospholipids (lysophosphatidylcholines, sphingomyelins, phosphatidylcholines, and phosphatidylethanolamines) and total acid phospholipids (monophosphoinositides, phosphatidylserines [44]. In an experimental investigation, Corazolum was injected intramuscularly to induce Corazolum-induced epileptoid seizures in rats. This was associated with a significant decrease in the phosphatidylcholine and elevation in lysophosphatidylcholines content. Blood samples were collected, centrifuged, and treated to remove the purified phospholipids by one-dimensional thin-layer chromatography. It was reported that cardiolipins and phosphatidylserines were significantly upregulated at the point of development of epileptic seizures, thereby confirming the essential level of respiratory potential at which the process of oxidation became much less rigorous in the ischemic brain. The study further determined the role of ultralow concentrations of vitamin E and sodium thiosulfate which were injected prior to Corazolum and observed that it had a mobilizing effect on the endogenous antiradical defense system of the cell. Disturbances in metabolism by the administration of Corazolum in phospholipids were induced in intact animals. A special feature observed during study of Corazolum-induced epileptic seizures was long-lasting benefits of lysophosphatidylcholines over the content of lysophosphatidylcholines in the control animals [44].

### 2.13. An Animal Model for Altered Tryptophan Metabolism Causing Myoclonus Epilepsy

Unverricht-Lundborg disease (ULD), also known as EPM1, is the most common form of progressive myoclonus epilepsy (PMEs) [117]. CSTB mutation is supposed to be the underlying cause of a majority of ULD cases which might ultimately lead to altered tryptophan metabolism followed by PME [117]. However, a new clinical and molecular form of ULD without mutation in CSTB gene has been reported [118]. The main features of EPM1 includes spontaneous, as well as stimulus-sensitive myoclonus, generalized tonic-clonic seizures, intention tremor, ataxia, incoordination, and dysarthria. However patients may progress to additional motor disabilities and even rapidly progressing dementia [119].

CSTB KO mice displays similar phenotypes of progressive neurodegeneration, ataxia and myoclonic seizures in a mammalian model of EPM1 mutation [120]. The study aimed to examine the tryptophan metabolism and the 5HT and KYN pathway in the brain of CSTB deficient mice in relation to their plausible involvement in seizure phenotype. The results showed that cerebral cortex and cerebellum of CSTB-deficient animal had elevated levels of 5HT, tryptophan, and 5-hydroxyindole acetic acid (5HIAA), as detected by high-pressure liquid chromatography (HPLC) assay. It was also reported that the level of KYN was increased in the cerebellum of CSTB-deficient mice. These neurotransmitter changes were associated with ataxia and myoclonic phenotype of epileptic seizures. The levels were increased due to deregulated tryptophan metabolism along the 5-HT and KYN pathways in the cerebellum of CSTB^−/−^ mice. The authors highlighted that CSTB mice provide a secondary enhancement for tryptophan metabolism in CNS which may contribute to epileptic-like seizures. The study should further be directed towards describing behavioral and chemical changes by mutating CSTB genes that affect the metabolism leading to epileptic seizures [45].

### 2.14. Long Noncoding RNAs (lncRNA) Cancer Susceptibility Candidate 2 (lncRNA CASC2) Inhibits Astrocytic Activation and Adenosine Metabolism

lncRNA are present in all types of living creatures with RNA transcripts that do not encodes proteins with a length of >200 nucleotides that do not encodes proteins [121]. Many biological phenomena demonstrate the involvement of lncRNAs, such as imprinting genomic loci, shaping chromosome conformation, and allosterically regulating enzymatic activity [122]. The implication of lncRNA genes has been found in numerous human diseases like overexpression, deficiency, or mutation. All the available lncRNAs majority of functions are not known, but many of those may not have good appreciable functions. The well understood few lncRNAs have their role to play in many diseased conditions such as X inactive specific transcript (XIST in X chromosome inactivation), HOX transcript antisense RNA (HOTAIR in positional identity) and telomerase RNA component (TERC in telomere elongation) [123]. The current research paper established a model of epilepsy in vitro and in vivo to investigate the biological role of lncRNAs CASC2. The behavioral test and expression of protein analysis proved that lncRNAs CASC2 overexpression improved the inhibition of epileptic seizures and astroglia activation. Regulation of phosphatase and tensin homolog (PTEN) in the brain by lncRNAs exerted an anti-epileptic effect. The study also found that PTEN and lncRNAs demonstrated downregulation during an epilepsy episode in hippocampal tissue and PTZ-evoked astrocytes. PTEN is positively correlated with lncRNA CASC2 expression. The overexpression of lncRNAs CASC2 mitigates hippocampal structural damage in rat brain by suppressing the astrocytes activation and adenosine metabolism during PTZ-induced epilepsy [46].

### 2.15. HMGB1 Modulates Glutamate Metabolism in KA-Induced Seizures

High mobility group box 1 (HMGB1) is an important chromatin protein that belongs to a damage-associated-molecular-patterns (DMAP) family [124,125]. The passive release of HMGB1 is observed from the nucleus in the extracellular space immediately after the neuronal injury. Inflammatory cell response during inflammation after the neuronal injury is initiated by HMGB1 and the process is activated by binding with the receptor for advanced glycation end-products (RAGE) and toll-like receptor 4 (TLR4) [126]. The episode of epilepsy is known to be contributed by HMGB1 protein that triggers tissue damage and inflammatory response via a TLR4-dependent pathway [127]. KA injection, as a glutamate agonist, to the excitatory neurotransmitter into the rodent brain induces epileptic seizures. Increased glutamate release into synaptic clefts and, in part, leads to excitotoxic lesion-induced epilepsy. KA has been reported to induce seizures in animals coincident with a significant increment in HMGB1 concentration in the hippocampus. The study reported the translocation of HMGB1 from nuclear to cytosol to extracellular space and the level of expression of glutamate metabolism enzymes. The primary rat neural cells (PRNCs) culture study, in which HMGB1 was acetylated and phosphorylated by KA-administration, reflecting its ability to function as an inflammatory mediator. The present results advance the concept of HMGB1 post-translational modifications, it contributes to the understanding of disease pathology and developing treatments for epilepsy-related hyperexcitability [47].

### 2.16. Lipid Metabolism Altered in Rat Model of Post-Traumatic Epilepsy (PTE)

Traumatic brain injury (TBI) also leads to epilepsy, known as PTE [128,129]. However, the percentage of people developing PTE after TBI is not well known. The occurrence of seizure mostly takes after few weeks or months of TBI in 1–5 of every ten people, depending on the location of the injury in the brain [130]. TBI is followed by neural inflammation, disruption of metabolism, and traumatized brain adding to the cause of an increase in oxidative stress, increase in free oxygen radicle, and hemoglobin disruption causing FE^2+^/FE^3+^ free ions. The research paper tried to explain the lipid metabolism in PTE condition of iron induced epileptic rat model. The in vitro study, high-resolution NMR and lipid staining studies were used to evaluate the parameters of altered lipid metabolism. Other aspects like the level of gene expression, cytokine release at the sight of insult, and enzyme activity were also evaluated. Epileptic seizures were examined by EEG and memory impairment was measured by behavioral studies. The altered lipid metabolism is observed in the brain tissue at a site of injection of FeCl_3_ which ultimately results in epileptic seizures after 30–45 days. The same phenomenon is observed in TBI patients having post-traumatic epilepsy. The focal epileptiform activity spreads from the site of origin to the whole cerebral cortex and numerous other brain structures. The study confirms the finding of lipid metabolism dysfunction in TBI to cause epileptic seizures in the later stage of life [130].

### 2.17. Altered Glycolysis and Mitochondrial Respiration in a Zebrafish Model of Dravet Syndrome (DS)

Severe myoclonic epilepsy of infancy (SMEI) was first described in 1978 in French and later in 1985 in English, and the name was changed to the DS in 1989. The mutation is carried by 70% of the population on the alpha subunit of the SCN1A gene [131]. The symptoms start from the first year of life with generalized or unilateral febrile clonic seizure in 64% of cases along with myoclonic jerk, with EEG showing generalized spike-waves and polyspike waves with photosensitivity and focal abnormality [132]. Retarded psychomotor development was observed from the second year of life, along with resistance to all forms of first line treatment. DS is also referred to as drug resistant epilepsy [133]. Increased energy consumption inside the brain is activated by neuronal activity and almost 20% of the total energy produced by body is used for various neuronal activities. In the epileptic event, there is an imbalance in the supply and demand chain, causing reduced energy production inside the brain [134]. The study demonstrated decrease in metabolism during epileptic seizure in zebrafish model of DS. The energy production was interrupted by decreasing glycolytic and mitochondrial respiration rates. The ketogenic diet (KD) has shown some positive results in the alteration of diet in DS patients and in Scn1aLab mutant zebrafish [135,136]. The authors claim to demonstrate the use of zebrafish as a model to study metabolic defects affecting epilepsy to evaluate role of chemoconvulsants or to identify compounds that modulate the process of glycolysis and energy production in disease states [49].

### 2.18. Alterations in Cytosolic and Mitochondrial [U-13C] Glucose Metabolism in a Chronic Epilepsy Mouse Model

Epileptic seizures are classified as idiopathic when the cause is genetic mutation or transmission contains epilepsy only with no external lesion [137]. Temporal lobe epilepsy shows high resistance to the treatment available and is a common form of adult epilepsy. The metabolic dysfunction in the brain tissue, such as glycolysis, the TCA cycle, and electron transport chain, contribute to the triggering and progression of epilepsy [138]. During the epileptic event, glucose consumption is increased in the brain whereas less glucose is utilized by the epileptic zone. I one of the animal studies with a lithium-pilocarpine rat model of epilepsy, the cerebral local glucose utilization rates were lowered, including the CA1 and CA3 hippocampal regions [139]. The current study tries to clear the exact changes that occur in the hippocampal glucose metabolism leading to the chronic epileptic stage. The study follows the ^13^C-glucose metabolism during the Pilocarpine-induced status epilepticus (SE) showing impairment to oxidative glucose metabolism along with TCA cycle enzymes deactivation. The reduced maximal activities of pyruvate dehydrogenase (PDH) and 2-oxoglutarate dehydrogenase in the hippocampal region decreases the ability to generate ATP in epileptogenic areas, which contributes to seizure development. This approach might represent a new therapies to inhibit seizures in the epileptic brain [50].

### 2.19. BAD KO Provides Metabolic Seizure Resistance in a Genetic Model of Epilepsy with Sudden Unexplained Death in Epilepsy

Apoptosis denotes programmed cell death, in which cell contents are packed into small membrane like structures for garbage collection by immune cells—it is also known as “programmed cell suicide”. Cellular apoptosis takes place via two mechanisms, namely the intrinsic pathway (heat, radiation, viral infection, hypoxia) and extrinsic pathway (TNF-induced model and FAS-ligand mediated model) [140]. The protein BCL2-associated agonist of cell death (BAD) is well-known for its role in cell apoptosis, but it is also reported to have a regulatory function in cell metabolism. The recent study has discovered that genetic KO of BAD gene (BAD KO) alters cellular metabolism in rat brain, thereby reducing its glucose consumption and increasing its ability to use ketone bodies for energy production [136,141]. The mice on KD diet show resistance to epileptic seizures in a BAD^−/−^ mice acute convulsant model. The idea behind the research was to evaluate the seizure protection ability of BAD ^−/−^ in a chronic epilepsy model such as Kcna1^−/−^ BAD^−/−^. The loss of function mutation of Kcna1 is a voltage gated potassium channel subunit Kv1.1 missense variant predominantly result into episodic ataxia1 and varied clinical conditions, including epilepsy, neuromyotonia, migraine, cognitive delay, etc. [142]. Kcna1^−/−^ Bad^−/−^ double knockout mice were generated by crossing Kcna1^+/−^ Bad^−/−^ mice with Kcna1^+/−^ Bad^−/−^ mice. The results suggest that BAD KO increases longevity and decreases seizure severity in Kcna1^−/−^ mice. The survival study of Kcn1^−/−^ mice showed that 80% die by week 5 of age but that all Kcna1^−/−^ BAD^−/−^ mice survived for a longer period. It was also found that Kcna1^−/−^ Bad^−/−^ mice showed less seizure frequency but could not survive a seizure score of 4/5. Thus, BAD KO mice extended longevity but could not prevent sudden unexplained/unexpected death in epilepsy (SUDEP) in a Kcna1^−/−^ model of epilepsy [51].

### 2.20. Metabolic Perturbations Associated with the Consumption of a Ketogenic Medium-Chain TAG Diet in Dogs with Idiopathic Epilepsy

The use of KD had been described in many studies and has officially been used to control seizures since 1920 as a non-medicated epileptic treatment on the basis of the alteration of daily diet. The study aims to identify the total metabolic changes linked with the administration of KD (medium-chain TAG diet) MCTD in dogs with idiopathic epilepsy (genetic mutations). The animal group was divided into fasting (placebo) and KD group with random male and female distribution. Various techniques like ultra-performance liquid chromatography-MS (UPLC-MS) were used to collect metabolic and lipidomic profiles of fasting dogs significant comparisons were made between MCTC and placebo diet phases. The results presented in this study show global changes in lipid metabolism and results of MCT consumption. The study also suggests that MCT consumption improves administration KD for neurological diseases, but also offers a new strategy for research to develop better therapy in the form of an epileptic diet [52].

### 2.21. A Novel Metabolism-Based Zebrafish Model to Uncovers HDACs 1 and 3 as a Potential Combined Anti-Seizure Drug Target

Epilepsy is a neurological disorder that commonly affects around ~1% global population with a varying etiology [143]. The rare genetic idiopathic epilepsy and most of the generalized form of epilepsy remains refractory to available treatment. It has been observed since the 1930s that there are more than 35 AEDs available and one third of the patients fail to respond to the treatment [144]. Thus, despite the arrival of newer drugs and years of research into the molecular neurology of epilepsy, the number of patients with uncontrolled epilepsy has remained essentially unchanged [145]. This study tries to report a new metabolic-based phenotypic drug screening model that can uncover novel targeted therapies relevant for future drug design. They found a consistency in phenotype resulting from pharmacological induction and targeted KO model. They screened 870 compounds and identified Vorinostat as a potent anti-seizure drug shown to demonstrate selective HDAC1 and HDAC3 inhibition. It was found to decrease seizure in Kcna1-null mice by 60% by using video-EEG recordings [53].

### 2.22. Pyridoxine-Dependent Epilepsy (PDE) in Zebrafish Caused by Aldh7a1 Deficiency

The rare metabolic epilepsy is caused by the accumulation of α-aminoadipic acid semialdehyde (α-AASA) and piperidine-6-carboxylic acid (PSC). The accumulation of this products takes place due to a mutation in ALDH7A1 gene encoding α-aminoadipic-semialdehyde dehydrogenase (α-AASAD) enzyme in the lysine catabolic pathway leading to PDE [146]. It is one of the rare autosomal recessive inherited metabolic disease in which neonatal or infantile seizures are increased uniquely by a high concentration of pyridoxine (pyr, Vitamin B6) or pyridoxal 5′-phosphate. Although PDE has been shown to be responsive to the pharmacological treatment of pyridoxin, it fails as a lifelong supplement by preventing neurodevelopment and causing disabilities observed in more than 75% PDE patients [147]. The paper describes the new technique of clustered regularly interspaced short palindromic repeat (CRISPR)/CAS9 gene editing to develop an aldh7a1-null zebrafish model. The mutation in zebrafish represented the clinical and biochemical characteristics of PDE. Aldh7a1 loss-of-function causes the accumulation of toxic PDE biomarkers, recurrent spontaneous seizures from day 10 post-fertilization (dpf), and premature death at day 14. When PDE zebrafish were treated with pyridoxin or PLP, it halts recurrent seizures and prolonged the survival of the mutated fish. The analysis technique of mass spectrometry (MS) of untreated aldh7a1 mutated fish identified a number of alterations in amino acid levels in the lysine metabolism pathway. Most importantly, they observed low B6 vitamers and gamma-aminobutyric acid (GABA) levels, which suggests that PED is at least a disorder of GABA homeostasis [54].

### 2.23. PDH Deficiency in Mouse Model

Glucose is the only source of energy in the brain which is the ultimate substrate for most brain activities that use carbon, including the synthesis of neurotransmitters. The energy production in the cell takes place via the oxidation of glucose in the mitochondrial TCA cycle. Before entry into the cycle, the pyruvate, which is formed due to glucose, must be transformed into acetyl coenzyme (acetyl-coA) in a reaction catalyzed by PDH. This conversation takes place in almost all the tissues in the body, but brain neuronal cells produce an extra two byproducts known as glutamate and GABA, sustaining and balancing most synaptic activity. The disorder in glucose metabolism is linked to disrupted excitability, and the clinical evidence includes a reduction in cerebral cortical activity, exemplified by PDH deficiency (PDHD). Most patients with PDHD harbor a mutation in the PDH α-subunit gene (PDHA1), which is transmissible in an X-linked fashion. The research paper tried to develop an animal model to better understand the pathophysiology of PDHD by generating mice with brain specific PDH activity that simultaneously shows the human disease features, including cerebral hypotrophy, decrease EEG amplitude, and epilepsy. The model developed was in mice with PDHA1 KO exhibiting a reduction in cerebral TCA cycle flux, glutamate production, and spontaneous and electrical evoked in-vivo cerebral field potential [55].

## 3. Materials and Methods

### 3.1. Search Methods

A comprehensive and systematic literature search was done summarizing the animal models available for ME. Numerous electronic databases were searched, namely Springer, Google scholar, PubMed, ScienceDirect, and Scopus from the period between 1 January 2000 and 30 April 2020. The search keywords used were “epilepsy”, “metabolism”, “metabolic epilepsy”, “metabolic epilepsy AND animal models”, and “metabolic epilepsy AND pre-clinical trials”.

### 3.2. Study Exclusion/Inclusion and Selection Criteria

The search strategy was only limited to original research articles that were published in English. Reviews, abstracts, books, chapters, patents, symposiums, oral and poster presentations, and conferences were excluded due to inadequate data for assessment and comparison with other studies. Moreover, articles that do not focus on ME were excluded. Articles which do not meet the review criteria, duplicate articles, and articles irrelevant to the aim of the review were also excluded. Studies were carefully chosen for insertion in the review that met the following inclusion criteria: (1) studies utilizing animal models to study ME, and (2) suggestive model mechanisms used effectively in pre-clinical and clinical studies of ME, (3) studies describing metabolic alterations related to epileptic disorder. Full text, original research articles related to ME only were included.

### 3.3. Data Extraction and Analysis

One researcher obtained data individually, and then abstracts and the titles of each research paper were compared under excel sheet to prevent the duplication of data. Based on the above-mentioned standard applied for searching, 151 full-text articles were assessed for eligibility, out of which 128 articles were discarded and 23 eligible research papers were assessed in this study (Figure 1). The prime purpose of using the PRISMA statement is to help authors to recognize and develop the reporting of systematic reviews and meta-analyses connected to the use of clinical and preclinical models in the study of metabolic epilepsy [148]. The chart and flow diagram were prepared according to PRISMA guidelines for the transparent reporting of systematic reviews and meta-analyses [149].

## 4. Conclusions and Future Direction

The development of a suitable and robust animal model that can mimic clinical phenotypes will be a noteworthy contribution in the domain of biomedical research. In a similar way, animal models can provide insights into the precise mechanisms associated with ME. Notably, an understanding of the possible basic and fundamental mechanism of ME is not solely dependent on the rodent models, but can also be studied in other models like zebrafish, dogs, drosophila, tilapia, and human models as these species have already scientifically proven their use in neurological research. It may be easier to study certain ME in human models such as reprogrammed neurons from patients rather than in rats. Different new gene editing techniques (CRISPR/CAS9) should be incorporated along with different analytical methods to find the basic molecular changes in the animal and human settings. This current study has systematically reviewed the available literature on experimental models that relate metabolic alterations due to epilepsy. Different animal models have emerged in recent years, which allows to study ME, but less attention has been paid to the development of a model of ME utilizing animals other than rodents, which are used currently. The review also highlights the critical need for the rationalization or regularization of animal models and evaluation methods to study ME. Moreover, the current review highlights the importance of animal models in ME, addressing the issues associated with translating the human clinical symptoms into animal settings. Future studies are recommended to validate the safety and efficacy of an animal model that would allow for the complete recapitulation of clinical symptoms of human ME.

## Figures and Tables

**Table 1:** Tabular representation of animal models in ME.

S.N.	Model Type	Study Type	Study Sample	Animal/Subject Used for Model Design	A Drug or Technique Used for Modeling Metabolic Epilepsy (ME)	Outcomes	No. of Citations	References
1	Metabolic gene responsible for epilepsy in obese rat	Pre-clinical (animal model)	*n* = 45 Groups—24 h (*n* = 5), 10 days (*n* = 5), 1month (*n* = 5) and 2 months (*n* = 5).	SD rats, 30–35 days old (125–150 g), male rats.	4% Pilocarpine hydrochloride (350 mg/kg in saline, i.p.), methyl-scopolamine prior to Pilocarpine Injection.	Abcc8 and Kcnj11 gene significantly different from control group. Hsd11b1 and Nr3c1 gene increase in fold change significantly different from control group. Metabolic dysfunction causes repeated reoccurrence of seizures leading to chronic epilepsy.	6	[33]
2	Metabolic profiling of epileptic rat brain (PTZ kindling induced seizures) Type: Creatine disorders and Succinic semialdehyde dehydrogenase deficiency	Pre-clinical (animal model)	*n* = 10	Male Wistar rats, group (a and b) were 4 weeks old and group (c) were 9 weeks.	PTZ (37 mg/kg body weight) every 48 h or every 72 h at the weekend over a 5-week period	Increase in metabolites like Myo-inositol, lactate, creatine, phosphocholine, GPC, Succinate. Clear metabolic changes in the cerebellum and hippocampus of kindled rats. Indicating increase in energy demand, altered neurotransmitters, increase neuron loss and gliosis. Technique used ^1^H NMR spectroscopy.	25	[34]
3	Mutation of ubiquitin ligase (Ube3a phenotype) causes Angelman syndrome in mice—a rare genetic epileptic neurodegeneration. Type: disruption of UBE3A, Mitochondrial disorders	Pre-clinical (animal model)	*n* = 6 LTP *n* = 11 LTP Maternal deficiency (*n* = 12)	UBE3Atm1Alb/J null mutation (AS) mice, UPD mice WT mice	8 and 12 weeks of age 12–16 weeks of age.	E6-AP protein can affect the metabolism of p53 in postmitotic neurons. Ube3a mice at maternal deficiency closely mimic the phenotype of human AS Decrease in GABA/Glutamate ratio following ketone ester administration.	608	[35,36]
4	Metabolic features in repetitive seizures. Type: Mitochondrial disorders	Pre-clinical (animal model)	(*n* = 7 immature; *n* = 6 mature)	Immature animals: SD rat pups (P15) Mature animals: male Sprague–Dawley rats (P60).	Flurothyl was used to induce repetitive seizures in immature and mature rats.	In immature rates, threshold of 2nd seizure was lower than for the 1^st^ seizure. I was vice versa for the mature rats. Immature animal showed more c-fos mRNA expression in the regions of the CNS. Consequences of repetitive seizures in immature animal is more related to metabolic disorder then mature animals.	10	[23]
5	Glut1 gene deficiency in mouse model. Type: GLUT-1 deficiency	Pre-clinical (animal model)	*n* = 104	G1D transgenic antisense mice 3 and 5 months of age	G1D gene knockdown to produce Glut1 deficiency	G1D antisense mice represents similar features that closely resemble human phenotype. Glutamine and its synthetase expression were preserved in G1D mice. TCA cycle intermediate, amino acid and neurotransmitter contents were normal, so there is no basis to suspect that in G1D mice, TCA cycle is responsible for energy failure.	16	[37]
6	Mitochondrial respiration deficits in rat epilepsy model Type: Mitochondrial disorders	Pre-clinical (animal model)	*n* = 4–8 in each group	Adult male SD rats (300–350 g)	KA (11 mg/kg, s.c.).	Mitochondrial respiration deficits occur in experimental. Novel methodology for assessing cellular metabolism. Increased steady-state ROS in mice and deletion of manganese superoxide dismutase results in deficits in mitochondrial oxygen consumption.	16	[38]
7	Abnormal metabolic function in the Pilocarpine-induced epilepsy rat model. Type: Mitochondrial disorders	Pre-clinical (animal model)		Adult male SD rats (7 weeks old), weighing 180–200 g	Pre-treated with lithium chloride (127 mg/kg, i.p.) & methylscopolamine-bromide (1 mg/kg, i.p) 24 h and 30 min before Pilocarpine administration. Pilocarpine hydrochloride (30 mg/kg, i.p.) was injected and repeated doses (10 mg/kg) were then administered every 30 min until stage 4 seizures developed according to the Racine scale	Metabolic connectivity was confirmed by magnetic resonance imaging (fMRI) based on blood-oxygen-dependent (BOLD) signals. The technique is used provide vital information on various brain disorders and functional connectivity abnormalities in diseases such as epilepsy. Complex brain connectivity, abnormal brain metabolism and topologic features were theoretically measured.	13	[39]
8	Metabolic dysfunction via adiponectin deficiency. Type: adiponectin -responsive seizures (Mitochondrial disorders)	Pre-clinical (animal model)	(*n* = 17 per genotype) (*n* = 10 per genotype)	C57BL/6J mice and ADP-KO mice. Control WT and Adiponectin-deficient mice	KA-Induced Seizure	Greater fat accumulation due to adiponectin deficiency. Low dose of intrahippocampal KA resulted in severe neuronal damage and gliosis in ADP-KO mice. Clonic seizures (seizure score of 3+) occurred in 50% of HFD-fed ADP-KO mice.	26	[40]
9	Myoclonus Epilepsy: impairment of serotonin (5HT) and 3-Hydroxyanthranilic Acid metabolism. Type: adiponectin-Responsive seizures (Mitochondrial disorders)	Pre-clinical (animal model) and Clinical study (Human Subjects)	*n* = 4 (mice) Unverricht- Lundborg type (EPM1) diagnosed human patients *n* = 2	The wild type mice (129SvJ strain) and heterozygous for CSTB mice age 4 months. Male and female age 35 ± 5 years	Valporic acid induced metabolic disturbances in myclonus epilepsy	Tryptophan metabolism along 5-HT and kynurenine (KYN) pathways are disrupted in EPM1. CSTB-deficient animals showed no change in tryptophan concentration. In humans’ patients with sodium valproate has been shown to reduce serum tryptophan level. Reduced absorption of tryptophan from GI tract is been observed with valproate treatment.	8	[41]
10	Model for metabolic dysfunction during epileptic seizure in Pilocarpine treated rats Type: Mitochondrial disorders	Pre-clinical (animal model) and Clinical study (Human subjects)	Rats *n* = 6	Male Wistar rats (115–130 g)	Pilocarpine hydrochloride (320 mg/kg, i.p.; 30 min after pre-treatment with scopolamine hydrobromide (1 mg/kg, s.c.; Pharmaco-resistant TLE involved patients.	Characterized metabolic and mitochondrial functions between acute hippocampal slices from epileptic rat’s brain and pharmaco-resistant TLE patients was done. NADPH transients were observed in dentate gyrus, CA3, CA1 of rat’s brain. The metabolic dysfunction elicited in each neuron of AHS tissues, represents a negative activation-dependent mitochondrial depolarization.	104	[42]
11	Lafora disease—altered glycogen metabolism causing epilepsy. Type: Laforin or malin deficency	Pre-clinical (animal model) In-vitro study	*n* = 3–8 genotype	Epm2a^−/−^ LKO mice model (mixed C57BL/6J and 129Sv/J)	Genetic knock down Epm2a^−/−^/Gys1^+/+^ are labeled as LKO mice model and Epm2a^−/−^/Gys1^+/+^ knock down are labelled as DKO experimental mice	Laforin or malin deficiency causes C6 hyperphosphorylation. Malformed long-chained glycogen gest collected in many brain cells causing epilepsy.	34	[43]
12	Phospholipid metabolic disorders- corazolum- induced seizures. Type: Phospholipid dysfunctioning	Pre-clinical (animal model)	*n* = 50	Male albino rats weighing 180–200 g,	Single intramuscularinjections of corazolum (dose, 8–9 mg peranimal), sodium thiosulfate (1 mg per animal), and vitamin E (0.4 mg per animal) to produce corazolum- induced seizures.	Decrease in t phosphatidylcholine and elevation in lysophosphatidylcholines content was observed. Cardiolipins and phosphatidylserines were significantly upregulated at the point of development of epileptic seizures.	0	[44]
13	Animal model for altered tryptophan metabolismin causing myoclonus Epilepsy. Type: Glutaric Aciduria	Pre-clinical (animal model)	*n* = 3	5-month-old mice homozygous. for a disruption in the Cstb gene (Cstb^−/−^, 129SvJ strain	By disruption in the Cstb gene	The CSTB-deficient mice had constitutively increased TRP, 5HT, and 5-hydroxyindole acetic acid (5HIAA) levels Increased levels of KYN in the cerebellum. CSTB metabolic gene deficiency in specific brain regions, may be responsible for the myoclonic/seizure.	14	[45]
14	long noncoding RNAs cancer susceptibility candidate 2 (lncRNA CASC2) inhibits astrocytic activation and adenosine metabolism	Pre-clinical (animal model)	5 group *n* = 12	Male SD rats (200−220 g).	LncRNA CASC2 suppression in PTZ induced rats.	Astrocytes activation is inhibited by LncRNA CASC2 in epileptic rats. Adenosine metabolism is inhibited by LncRNA CASC2 in the epileptic rat’s hippocampus. Adenosine metabolism-related proteins p-P38, ENT1 and ADK were also found to be reduced in PTZ treated rats, which were increased by lncRNA CASC2.	1	[46]
15	HMGB1 modulates glutamate metabolism in KA induced seizures	Pre-clinical (animal model)	Neuronal cell culture plate—Cells (4 × 104 cells/well)	Primary rat neural cells (PRNCs)—BrainBit (E18 rat cortex)	KA—10 μM	Translocation of HMGB1 from nuclear to cytosol to extracellular space. HMGB1 contributed protein that triggers tissue damage and inflammatory response.	13	[47]
16	Lipid metabolism altered in post-traumatic epileptic rat model	Pre-clinical (animal model)	2 groups (*n* = 10; *n* represents the number)	Six months old male Wistar rats, weighing 350–400 g	ferric chloride (FeCl3) to cause post-traumatic epilepsy (PTE).	Lipid peroxidation causes selective alteration in cell signaling, protein and DNA damage and cytotoxicity in damaged brain. Free iron radicals Fe^2+^/Fe^3+^ from damaged hemoglobin induces inflammatory mechanism at the accident sight. Oxidative-stress causes peroxidation of lipids which induced damage or destruction of lipid components in the brain.	4	[48]
17	Altered glycolysis and mitochondrial respiration in a zebrafish model of Dravet Syndrome	Pre-clinical (animal model)	96 plate well	Scn1Lab mutant zebrafish (HM/WT), 5dfp	voltage-gated sodium channel-1A_Lab mutation (SCN1A_Lab)	Scn1Lab mutant zebrafish showed a decrease in baseline glycolytic rate and oxygen consumption rate (OCR) Glucose and mitochondrial hypometabolism contribute to the pathophysiology of Dravet Syndrome.	24	[49]
18	Alterations in cytosolic and mitochondrial [U-13C] glucose metabolism in a chronic epilepsy mouse model	Pre-clinical (animal model)	*n* = 10–12 group -2	Male CD1 mice	Pilocarpine induced status epilepticus (SE) model	The metabolic dysfunction such as glycolysis, the TCA cycle and electron transport trigger epilepsy. Impairment to oxidative glucose metabolism along with TCA cycle enzymes deactivation is observed in epileptic brain.	9	[50]
19	BAD KO provides metabolic seizure resistance in a genetic model of epilepsy with SUDEP	Pre-clinical (animal model)	Male and female Kcna1^−/−^ (*n* = 29; 10 female, 19 male) and Kcna1^−/−^ Bad^−/−^ (*n* = 15; 10 female, 5 male) mice	Kcna1^−/−^ mice	BCL2-associated agonist of cell death (BAD)—Kcna1^−/−^ mice	BAD KO increases longevity and decreases seizure severity in Kcna1^−/−^ mice. Kcna1^−/−^ Bad^−/−^ mice outlived Kcna1^−/−^ mice by approximately 2 weeks. Kcna1^−/−^ Bad^−/−^ mice also spent significantly less time in seizure than Kcna1^−/−^ mice on P24.	6	[51]
20	Metabolic perturbations associated with the consumption of a ketogenic medium-chain TAG diet in dogs with idiopathic epilepsy	Pre-clinical (animal model)	Male *n* = 10 and female *n* = 6 dogs Avg. weight 29.3 kg Avg. year 4.59 years old	21 dogs with idiopathic epilepsy of different breed	Idiopathic epilepsy in dogs	To identify the total metabolic changes linked with the administration of ketogenic diet (medium-chain TAG diet) MCTD in dogs. Various techniques like ultra-performance liquid chromatography-MS (UPLC-MS) were used to collect metabolic and lipidomic profiles. The study also suggests that MCT consumption improves administration of ketogenic diet for neurological diseases but also offers new strategy for research.	8	[52]
21	A novel metabolism-based zebrafish model to uncovers HDACs 1 and 3 as a potential combined anti-seizure drug target:	Pre-clinical (animal model)	Zebrafish larvae, Kcna1-null mice	5–7dpf 96 plate well, wild-type zebrafish (TL strain) Kcna1-null mice	Kcna1-null mice, PTZ induced zebrafish model.	Study tries to report the new metabolic based phenotypic drug screening model that can uncover novel targeted therapy relevant for future drug design. They found consistency in phenotype resulted from pharmacological-induction and targeted KO model. They screened 870 compounds and identified Vorinostat as a potent anti-seizure drug and showed to have a selective HDAC1 and HDAC3 inhibition.	15	[53]
22	Pyridoxine-dependent epilepsy in zebrafish caused by Aldh7a1 deficiency	Pre-clinical (animal model)	Zebrafish larvae	Zebrafish larvae 5–14dpf	Aldh7a1-null mutation, pyridoxin dependent epilepsy	The paper describes the new technique of clustered regularly interspaced short palindromic repeat (CRISPR)/CAS9 gene editing. Aldh7a1 loss-of-function cause accumulation of toxic PDE biomarkers, recurrent spontaneous seizures from day 10 post-fertilization (dpf) and premature death at day 14. The analysis technique like mass spectrometry (MS) of untreated aldh7a1 mutated fish identified number of alterations in amino acid levels, lysine metabolism pathway.	32	[54]
23	Pyruvate dehydrogenase deficiency in mouse model	Pre-clinical (animal model) Clinical data	Human blood sample mouse model of (PDHD)	Zebrafish larvae and Pdha1 KO mouse 2–3 months old	Pdha1 knockdown mouse model (PDHD)	PDHD mice exhibited decreased cerebral glutamate concentration but normal GABA content. EEG recordings from the mice and patients with PDHD confirmed globally decreased basal electrical activity.	3	[55]

## Abbreviations

5-HT	Serotonin
CNS	Central Nervous System
ILAE	International League Against Epilepsy
5MTHF	5-Methyltetrahydrofolate
CFD	Cerebral Folate Deficiency
GAMT	Guanidinoacetate Methyltransferase
AGAT	Arginine Glycine Amidino Transferase
PNPO	Pyridoxamine Phosphate Oxidase
CFD	Cerebral Folate Deficiency
FOLR	Folate Receptor 1
GLUT1	Glucose Transporter 1
MERRF	Myoclonic Epilepsy with Ragged Red Fibers
MELAS	Mitochondrial Encephalopathy with Lactic Acidosis and Stroke-like Episodes
BDNF	Brain Derived Neurotropic Factor
TCA	Tricarboxylic Acid Cycle
ATP	Adenosine Triphosphate
HNMR	Proton Nuclear Magnetic Resonance
PTZ	Pentylenetetrazol
KA	Kainic Acid
LTP	Long Term Potentiation
ROS	Reactive Oxygen Species
fMRI	Functional Magnetic Resonance Imaging
HFD	High Fat Diet
CSTB	Cystatin B Gene
LD	Lafora Disease
TCA	Tricarboxylic acid
5HIAA	5-Hydroxyindole Acetic Acid
Ube3A	Ubiquitin-protein ligase E3A
PED	Pyridoxine-Dependent Epilepsy
HMGB1	High Mobility Group Box 1
CRISPR	Clustered Regularly Interspaced Short Palindromic Repeats
Cas9	CRISPR Associated Endonuclease 9
HDAC 1-3	Histone Deacetylases
Aldh7a	Aldehyde Dehydrogenase (ALDH) 7 Family Member A1 gene
Scn1a	Sodium Voltage-Gated Channel Alpha Subunit 1
α-AASAD	α-Aminoadipic-Semialdehyde Dehydrogenase
PSC	Piperidine-6-Carboxylic Acid
PCDHA1	Pyruvate Dehydrogenase α-Subunit Gene
GABA	α-Aminobutyric Acid
KCNA1	Potassium Voltage-Gated Channel Subfamily A Member1
EEG	Electroencephalogram
TBI	Traumatic Brain Injury
KD	Ketogenic Diet
SMEI	Severe Myoclonic Epilepsy of Infancy
RAGE	Receptor For Advanced Glycation End-products
TLR4	Toll-Like Receptor- 4
PRNCs	Primary Rat Neural Cells
SLC2A1	Solute Carrier Family 2 Member1
SD	Sprague Dawley
